# From simulation research to education policy: how much evidence is enough?

**DOI:** 10.1186/s41077-016-0023-0

**Published:** 2016-06-23

**Authors:** Ryan Brydges

**Affiliations:** 1 University of Toronto, Toronto, Ontario Canada; 2 Department of Medicine, University of Toronto, Toronto, Canada; 3 The Wilson Centre, University Health Network, Toronto, Canada

Most researchers aim to conduct studies that generate new knowledge for their field. In many countries, the twenty-first century brought with it a powerful discourse demanding research serve a dual purpose: researchers should aim to generate and translate knowledge. Hence, the research community has been assigned the task of balancing the science of discovery with the science of application [1]. Consequently, researchers now consider the implications of their work for health professions education curricula, healthcare systems, and healthcare policy. Applied work in implementation science and knowledge translation has become a field unto itself, and the state of evidence is captured well in the quote: “Though the problems translating or applying research in policy-making are legion, solutions are rare” [2]. A key question in this literature is what level of evidence is required to initiate translations from research to policy-making?

How has this tension between discovery and application emerged in the study of technology-enhanced and simulation-based learning in healthcare? The examples are many, so my focus here is on a recent longitudinal, multi-institutional study with significant implications: the National Council of State Boards of Nursing (NCSBN) National Simulation Study [3]. Over 2 years, the researchers obtained data from 666 nursing students representing 10 prelicensure programs in the USA. They randomized students to three experimental groups: a “traditional” control group (<10 % of clinical hours spent in simulation), a 25 % group (25 % of clinical hours replaced with simulation hours), and a 50 % group (50 % of clinical hours replaced with simulation hours). Key findings included no statistically significant differences between groups in nursing knowledge (measured by the ATI RN Comprehensive Predictor^®^ 2010 and National Council Licensure Examination (NCLEX^®^)), in clinical competency at graduation (measured by clinical preceptors’ in simulation and clinical contexts), or in managers’ ratings of clinical competency and readiness for practice at 6 weeks, 3 months, and 6 months in practice as a registered nurse (all *p* > 0.4). The authors interpreted the evidence as substantial, suggesting a well-designed simulation can replace up to 50 % of traditional clinical nursing training; an impressive study in scale, effort, and implications.

Changes resulting from the National Simulation Study have been considerable. Eight months following publication, a panel convened at the 2015 International Nursing Association for Clinical Simulation and Learning (INACSL) conference where the study results were well-received by the nursing community, yet concerns remained particularly around the faculty development that would be needed to ensure high-quality simulation training across institutions [4]. In close proximity to this conference, the NCSBN also convened an expert panel. Representatives from organizations including INACSL, the American Association for Colleges of Nursing, the National League for Nursing, and the Society for Simulation in Healthcare discussed the study alongside the broader nursing simulation literature and the INACSL Standards of Best Practice: Simulation^SM^ [5]. The panel produced a set of national simulation guidelines for prelicensure nursing programs in the USA. In November 2015, Pamela Jeffries presented the study findings at the Simulation Summit (Banff, Alberta, Canada) and noted that many American states are taking steps to adopt these guidelines as policy for prelicensure nursing training. Remarkable progress in remarkable time.

Is one study, no matter how large, enough to prompt such policy change? Was the single study in this case the spark the grassroots nursing community was waiting for or one that simulation interest groups wished for? At danger of wading into the weeds, a close inspection of the NCSBN study leads to a number of questions: should this superiority study design be followed up with, perhaps, more appropriate, non-superiority, and equivalence trials [6]? Did the authors consider evidence that variation in performance on large knowledge exams, like the NCLEX^®^, is accounted for most by individual student differences and least by curricula and educational policies [7]? While the measures of clinical competency have favorable reliability evidence, is the nursing community aware that much more validity evidence is recommended before using any assessment results to justify such high-stakes decision-making [8]? Clearly, there are a number of questions requiring a series of studies be conducted in this important area.

How much evidence is “enough” when it comes to policy change? If we take clinical practice as an example, recommended changes in clinical strategies and patient care require high-quality evidence generated in multiple randomized controlled trials [9]. In most cases, clinical guidelines are built on pre-existing knowledge and are not changed without comprehensive literature reviews and consensus-building meetings [10]. A single study hardly ever leads to policy change, except in the rare case that it tips the balance of evidence. The policy changes to how nursing schools incorporate simulation-based training into their curricula are based on a single study and two consensus-building processes. While the NCSBN study is impressive, researchers still must conduct additional studies to produce further evidence, judge the quality of evidence as it accumulates, and resolve implementation issues (e.g., faculty development).

Leaping out of the weeds, let us return to the overarching discourse of discovery versus application. As a community, simulation educators and researchers will grapple with this tension for years to come. We want to do our best in both regards. We are studying simulation as a means to discover the mechanisms of learning in healthcare professionals and trainees, while also studying simulation as a modality we can integrate as a component of meaningful, effective, and efficient curricula. As the community continues to conduct higher quality research, the implications for education and healthcare policy cannot be denied. Yet we must be cautious in how we translate our evidence. Much like we have adopted research principles and strategies from other fields—like social sciences, psychology, education science, and quality improvement—now appears to be a time for us to do the same via collaboration and rigorous research when we engage in implementation science and knowledge translation.

## References

[1] Wapner J. The false distinction between basic and applied science. http://blogs.plos.org/workinprogress/2011/07/24/the-false-distinction-between-basic-and-applied-science. Accessed on 7 May 2016.

[2] Trostle J, Bronfman M, Langer A (1999). How do researchers influence decision-makers? Case studies of Mexican policies. Health policy and planning.

[3] Hayden JK, Smiley RA, Alexander M, Kardong-Edgren S, Jeffries PR (2014). The NCSBN National Simulation Study: a longitudinal, randomized, controlled study replacing clinical hours with simulation in prelicensure nursing education. Journal of Nursing Regulation.

[4] Rutherford-Hemming T, Lioce L, Jeffries PR, Sittner B (2016). After the National Council of State Boards of Nursing Simulation Study—recommendations and next steps. Clinical Simulation in Nursing.

[5] Alexander M, Durham CF, Hooper JI, Jeffries PR, Goldman N, Kardong-Edgren S, Kesten KS, Spector N, Tagliareni E, Radtke B, Tillman C (2015). NCSBN simulation guidelines for prelicensure nursing programs. Journal of Nursing Regulation.

[6] Tolsgaard MG, Ringsted C (2014). Using equivalence designs to improve methodological rigor in medical education trials. Med Educ..

[7] Hecker K, Violato C (2008). How much do differences in medical schools influence student performance? A longitudinal study employing hierarchical linear modeling. Teaching and learning in medicine.

[8] Cook DA, Brydges R, Ginsburg S, Hatala R (2015). A contemporary approach to validity arguments: a practical guide to Kane's framework. Medical education.

[9] Anonymous. Applying class of recommendations and level of evidence to clinical strategies, interventions, treatments, or diagnostic testing in patient care https://eccguidelines.heart.org/index.php/tables/applying-class-of-recommendations-and-level-of-evidence-to-clinical-strategies-interventions-treatments-or-diagnostic-testing-in-patient-care. Accessed on 7 May 2016.

[10] Jaeschke R, Guyatt GH, Dellinger P, Schünemann H, Levy MM, Kunz R, Norris S, Bion J (2008). Use of GRADE grid to reach decisions on clinical practice guidelines when consensus is elusive. BMJ..

